# Supplementary material for: Are Higher Body Mass Index and Worse Metabolic Parameters Associated with More Aggressive Differentiated Thyroid Cancer? A Retrospective Cohort Study
Source: Healthcare (Basel). 2024 Mar 2;12(5):581. doi: 10.3390/healthcare12050581 (PMC10930676; doi:10.3390/healthcare12050581)
Supplement: Supplementary file 1 [file healthcare-12-00581-s001.zip › healthcare-2877475-supplementary proof done- UY 2-3-24.pdf]

Table S1- Treatment modalities and follow-up data of patients with DTC, according to their BMI group[†]

		Normal weight<br>BMI 18.5-24.9<br>N=47, 28.5%	Overweight and obese<br>BMI ≥ 25<br>N=118, 71.5%	P value
Thyroid surgery (n,%)	Partial	18, 38.3%	24, 20.34%	0.017
	Complete	29, 61.7%	94, 79.66%	
RAI treatment (n,%)	No	24, 51.06%	47, 39.83%	0.272
	30 mCi	5, 10.64%	23, 19.49%	
	≥100 mCi	14, 29.79%	38, 32.2%	
Risk assessment[¥] (n,%)	Low	27, 57.45%	54, 45.76%	0.21
	Intermediate or high	16, 34.04%	54, 45.76%	
Response to treatment[§] (n,%)	Excellent	36, 76.6%	76, 64.41%	0.149
	Incomplete[#]	3, 6.38%	16, 13.56%	
Additional intervention[@] (n,%)		0, 0%	8, 6.78%	0.106

[†]Sub-group analysis of patients whose age was 30-70 years at the time of the index thyroid surgery; a comparison of two BMI groups- normal weight versus overweight and obese; [¥]Risk of structural disease recurrence; [§]Response to treatment at the end of follow-up; [#]Biochemical and/or structural[^]; [@]Second thyroid operation, and/or neck dissection, and/or second RAI treatment, and/or external beam irradiation; Due to missing data in some variables the total percent per column might not summarize to 100%.
Abbreviations: DTC- differentiated thyroid carcinoma, BMI- body mass index (body weight [Kg] divided by the squire of height [meters]), N- number, RAI- radioactive iodine, mCi- millicurie

[^]Haugen BR, Alexander EK, Bible KC, Doherty GM, Mandel SJ, Nikiforov YE, Pacini F, Randolph GW, Sawka AM, Schlumberger M, Schuff KG, Sherman SI, Sosa JA, Steward DL, Tuttle RM, Wartofsky L. 2015 American Thyroid Association Management Guidelines for Adult Patients with Thyroid Nodules and Differentiated Thyroid Cancer: The American Thyroid Association Guidelines Task Force on Thyroid Nodules and Differentiated Thyroid Cancer. Thyroid. 2016 Jan;26(1):1-133. doi: 10.1089/thy.2015.0020. PMID: 26462967; PMCID: PMC4739132.

Table S2- Histopathological features (full description) following thyroid surgery of patients with DTC, according to their BMI group

			Normal weight BMI 18.5-24.9 N=66 , 31.3%	Overweight BMI 25-29.9 N=81 , 38.4%	Obesity BMI ≥30 N=64 , 30.3%	P value*
**Tumor**	pT (n,%)	pT 1-2	55, 83.33%	63, 77.78%	50, 78.13%	* 0.763<br>a 0.484<br>b 0.563<br>c 0.929
		pT 3-4	11, 16.67%	17, 20.99%	13, 20.31%	
	Histological sub-type (n,%)	PTC	60, 90.91%	75, 92.59%	60, 93.75%	* 1<br>a 1<br>b 1<br>c 1
		FTC	5, 7.58%	6, 7.41%	4, 6.25%	
	PTC variant (n,%)	Classic	32, 48.48%	25, 30.86%	20, 31.25%	* 0.62<br>a 0.6<br>b 0.332<br>c 0.69
		Follicular	24, 36.36%	41, 50.62%	30, 46.88%	
		Aggressive[¥]	4, 6.06%	9, 11.11%	10, 15.63%	
	Multifocality (n,%)	No	39, 59.09%	43, 53.09%	40, 62.5%	* 0.486<br>a 0.403<br>b 0.771<br>c 0.255
		Yes	26, 39.39%	38, 46.91%	24, 37.5%	
	Two lobes involvement (n,%)	No	39, 59.09%	43, 53.09%	40, 62.5%	* 0.642<br>a 0.347<br>b 0.608<br>c 0.669
		Yes	15, 22.73%	24, 29.63%	19, 29.69%	
	Extrathyroidal extension (n,%)	None	51, 77.27%	57, 70.37%	43, 67.19%	* 0.45<br>a 0.6<br>b 0.035<br>c 0.027
		Microscopic	9, 13.64%	14, 17.28%	19, 29.69%	
		Gross	5, 7.58%	9, 11.11%	1, 1.56%	
	Vascular invasion (n,%)	No	50, 75.76%	67, 82.72%	53, 82.81%	* 0.533<br>a 0.383<br>b 0.304<br>c 0.822
		Yes	15, 22.73%	14, 17.28%	10, 15.63%	
**LN**	LN MTS (n,%)	No	52, 78.79%	59, 72.84%	41, 64.06%	* 0.349<br>a 0.372<br>b 0.129<br>c 0.576
		N1a	6, 9.09%	14, 17.28%	13, 20.31%	
		N1b	7, 10.61%	8, 9.88%	9, 14.06%	
	Bilateral lateral neck LN MRS (n,%)	No	66, 100%	79, 97.53%	61, 95.31%	* 0.233<br>a 0.502<br>b 0.116<br>c 0.655
		Yes	0, 0%	2, 2.47%	3, 4.69%	

	>5 LN MTS[§] (n,%)	No	59, 89.39%	73, 90.12%	54, 84.38%	* 0.529<br>a 0.884<br>b 0.396<br>c 0.297
		Yes	7, 10.61%	8, 9.88%	10, 15.63%	
	LN diameter≥3 cm[#] (n,%)	No	64, 96.97%	81, 100%	60, 93.75%	* 0.037<br>a 0.2<br>b 0.437<br>c 0.036
		Yes	2, 3.03%	0, 0%	4, 6.25%	
	Extra-nodal extension[#] (n,%)	No	61, 92.42%	72, 88.89%	55, 85.94%	* 0.436<br>a 0.754<br>b 0.204<br>c 0.46
		Yes	4, 6.06%	7, 8.64%	8, 12.5%	
**Distal MTS[#] (n,%)**		No	61, 92.42%	79, 97.53%	63, 98.44%	* 0.134<br>a 0.407<br>b 0.119<br>c 0.504
		Yes	4, 6.06%	2, 2.47%	0, 0%	

Abbreviations: DTC- differentiated thyroid carcinoma, BMI- body mass index (body weight [Kg] divided by the squire of height [meters]), N- number, PTC- papillary thyroid carcinoma, FTC- follicular thyroid carcinoma, LN- Lymph node/s, MTS- Metastasis

* Comparison of all 3 sub-groups, a- overweight versus normal weight, b-Obese versus normal weight, c- obese versus overweight; [¥]Oncocytic, tall cell, insular, moderately differentiated, and poorly differentiated variants; § Central (level 6) and/or Lateral (levels 1-5); [#]One or more
Due to missing data in some variables the total percent per column might not summarize to 100%.

Table S3- Association between fasting glucose as a continuous variable and selected histopathological features

histopathological feature	Sub-categories	Unadjusted Odds Ratio (95% CI)*	Adjusted@ Odds Ratio (95% CI)*
Histological sub-type and variants	PTC- classic variant	1.00 (REF)	1.00 (REF)
	PTC- Follicular variant	1 (0.988-1.013)	0.996 (0.983-1.008)
	Aggressive variant¥ of PTC and/or FTC	1.001 (0.985-1.017)	0.992 (0.973-1.011)
Extrathyroidal extension	None	1.00 (REF)	1.00 (REF)
	Microscopic	0.998 (0.983-1.014)	0.996 (0.978-1.013)
	Gross	1.003 (0.985-1.022)	0.999 (0.979-1.02)
Vascular invasion	No	1.00 (REF)	1.00 (REF)
	Yes	0.988 (0.967-1.01)	0.986 (0.963-1.009)
Multifocality	No	1.00 (REF)	1.00 (REF)
	Yes	0.999 (0.987-1.01)	0.997 (0.985-1.009)
Lateral neck LN MTS	No	1.00 (REF)	1.00 (REF)
	Yes	1.002 (0.971-1.035)	1.001 (0.967-1.037)
LN MTS ≥ 3 cm	No	1.00 (REF)	1.00 (REF)
	Yes	1 (0.965-1.037)	1.004 (0.971-1.039)
Extra-nodal extension	No	1.00 (REF)	1.00 (REF)
	Gross	1.004 (0.988-1.021)	1.004 (0.986-1.023)
Distal MTS	No	1.00 (REF)	1.00 (REF)
	Yes	1.006 (0.98-1.033)	1.001 (0.969-1.034)

@Adjusted for age, sex, and ethnicity; *Bolded for p≤0.05; ¥ Tall cell, columnar, and insular variants of PTC together with moderately and poorly differentiated variants.
Abbreviations: CI- confidence interval, PTC- papillary thyroid carcinoma, FTC- follicular thyroid carcinoma, LN- Lymph node/s, MTS- Metastasis

Table S4- Association between triglyceride level as a continuous variable and selected histopathological features

Clinical/ histopathological feature	Sub-categories	Unadjusted Odds Ratio (95% CI)*	Adjusted@ Odds Ratio (95% CI)*
Histological sub-type and variants	PTC- classic variant	1.00 (REF)	1.00 (REF)
	PTC- Follicular variant	1.001 (0.997-1.006)	1.001 (0.996-1.006)
	Aggressive variant¥ of PTC and/or FTC	1.005 (1-1.011)	1.004 (0.998-1.01)
Extrathyroidal extension	None	1.00 (REF)	1.00 (REF)
	Microscopic	1.002 (0.997-1.006)	1.001 (0.996-1.006)
	Gross	0.996 (0.988-1.005)	0.993 (0.984-1.003)
Vascular invasion	No	1.00 (REF)	1.00 (REF)
	Yes	0.999 (0.993-1.004)	0.998 (0.993-1.004)
Multifocality	No	1.00 (REF)	1.00 (REF)
	Yes	1.001 (0.997-1.005)	1 (0.996-1.004)
Lateral neck LN MTS	No	1.00 (REF)	1.00 (REF)
	Yes	0.996 (0.981-1.011)	0.991 (0.974-1.009)
LN MTS ≥ 3 cm	No	1.00 (REF)	1.00 (REF)
	Yes	1.007 (0.996-1.017)	1.005 (0.994-1.017)
Extra-nodal extension	No	1.00 (REF)	1.00 (REF)
	Yes	1.001 (0.994-1.008)	0.999 (0.991-1.007)
Distal MTS	No	1.00 (REF)	1.00 (REF)
	Yes	0.997 (0.981-1.013)	0.994 (0.977-1.012)

@Adjusted for age, sex, and ethnicity; *Bolded for p≤0.05; ¥Tall cell, columnar, and insular variants of PTC together with moderately and poorly differentiated variants.

Abbreviations: CI- confidence interval, PTC- papillary thyroid carcinoma, FTC- follicular thyroid carcinoma, LN- Lymph node/s, MTS- Metastasis
